# Coronal Imbalance after Selective Posterior Thoracic Fusion in Patients with Lenke 1 and 2 Adolescent Idiopathic Scoliosis

**DOI:** 10.1155/2018/3476425

**Published:** 2018-12-09

**Authors:** Heng Jiang, Wei Shao, Enjie Xu, Zhe Ji, Tao Lin, Yichen Meng, Jun Ma, Ce Wang, Rui Gao, Xuhui Zhou

**Affiliations:** Department of Orthopedics, Changzheng Hospital, Second Military Medical University, Shanghai, China

## Abstract

Coronal decompensation is a common complication in Lenke 1 or 2 AIS patients after selective thoracic fusion (STF). However, the majority who developed immediately postoperative coronal decompensation experienced improvement and the related factors are not fully understood. The aim of this retrospective study was to investigate the prevalence of coronal imbalance in patients with Lenke 1 or 2 AIS and to explore radiological factors associated with spontaneous correction of coronal balance after surgery. Lenke 1 or 2 AIS patients receiving STF in our center from January 2013 to March 2015 were analyzed. Anteroposterior and lateral films were evaluated before surgery, at 1 month's and 2 years' follow-up. Patients were divided into 2 groups according to whether coronal imbalance occurred in the early postoperative period (1 month). Various radiological parameters as well as Scoliosis Research Society-22 were statistically compared between groups. Coronal decompensation was observed in 33 patients preoperatively, in 48 patients immediately postoperatively, and in 2 patients at final follow-up. Lowermost instrumented vertebra (LIV) disc angle (0.9° vs. 6.7°, p=0.019) and LIV- C7 plumb line and central sacral vertical line (CSVL) (-3.4mm vs. -13.7mm, p=0.020) increased in the final follow-up in the imbalanced group of type A modifier. The magnitude of lumbar curve was greater in the imbalanced group of type B or C modifier in the early postoperative period (19.5° vs. 12.6°, p=0.006; 25.5° vs. 13.7°, p<0.01), and this difference disappeared in the final follow-up. No differences in SRS-22 outcome scores were noted between groups in different time. Coronal imbalance was frequently detected immediately after STF in Lenke 1 or 2 AIS patients, with type C modifier slightly higher than A or B. Distal adding-on may help compensate for coronal imbalance in patients with type A modifier, while spontaneous correction of lumbar curve attributes to the improvement of coronal imbalance in patients with type B or C modifier.

## 1. Introduction

Selective thoracic fusion (STF) for Lenke 1 and 2 adolescent idiopathic scoliosis (AIS) patients was a generally accepted procedure. However, in some patients, immediately postoperative and late decompensation of coronal balance have been noted [1]. Mild coronal balance could be well tolerated, but patients with severe coronal balance need reoperation and tend to have inferior self-assessment/satisfaction with treatments [2].

Many scholars have studied this common phenomenon after selective deformity correction and numerous causes have been reported including preoperative decompensation [3], lowest instrumented vertebra (LIV) selection relative to stable vertebra (SV) [4], overcorrection of thoracic curve [5], and incorrect use of STF [6]. Interestingly, many patients who developed coronal imbalance in the early postoperative period experience improvement of coronal balance, and few patients persist to have coronal decompensation [4, 7]. However, there have been rare reports focusing on the spontaneous correction of coronal balance or its mechanism in Lenke 1 and 2 AIS patients.

Thus, the purposes of this study were (1) to illustrate the distribution and transition of coronal balance in Lenke 1 and 2 curves with different lumbar curve modifiers and (2) to reveal the related factors of the improvement of coronal balance.

## 2. Materials and Methods

The research project was approved by the Ethics Department of Shanghai Changzheng Hospital, Shanghai. We have consensus with all participants. All the procedures were done under the Declaration of Helsinki and relevant policies in China. This study was a retrospective study. A total of consecutive 136 patients with Lenke 1 and 2 AIS who underwent corrective surgery performed by a single surgeon at a single center between January 2013 and December 2015 was enrolled in this study. Inclusion criteria were as follows: (1) selective thoracic fusion with pedicle screw system of LIV ending at L1 or above for Lenke 1/2 AIS and (2) patients with minimum 2 years follow-up. All patients were regularly followed up at 1, 6, and 12 months after surgery and yearly thereafter. Demographic data including sex, age, and menarche status were obtained from electronic medical records. Pre- and postoperative SRS-22 scores were used to access clinical outcomes.

### 2.1. Radiological Measurements

Radiological parameters were measured in whole-spine standing anteroposterior and lateral radiographs preoperatively, 1 month after operation and at final follow-up. The magnitude of the curve was measured by using Cobb's angle method. Apical vertebral translation (AVT) was defined as the distance between the center of apical vertebra and central sacral vertical line (CSVL). Uppermost instrumented vertebra (UIV) tilt was measured using the angle between the upper endplate of UIV and the horizontal line. LIV tilt was measured using the angle between the lower endplate of LIV and the horizontal line. The LIV-CSVL distance was defined as the linear distance from the CSVL to the centroid of the LIV. LIV disc angle was measured using the angle between the upper and lower endplate of the first disc below LIV.

Coronal imbalance was defined as a >20 mm distance between the C7 plumb line and CSVL. Coronal balance and AVT were defined as a negative value when the C7 plumb line or the center of apex for MT/TL curves locates at the left side to the CSVL. The patients with a certain lumbar curve modifier were divided according to the presence or absence of coronal imbalance in the early postoperative period (a month postoperatively) into the imbalanced group and balanced group.

### 2.2. Surgical Protocol

All patients underwent posterior STF. All surgeries were conducted by a single surgeon at a single center. Rod rotation was taken as corrective maneuver. Pedicle screws were used in all cases, with additional attachment of some hooks in some patients. For STF, selection of the LIV was the last vertebra significantly touched by the CSVL and selection of the UIV was guided by the supine side bending film. Both somatosensory-evoked and motor-evoked potentials' neurological monitoring was used throughout operation.

### 2.3. Statistical Analyses

Demographic data were analyzed descriptively. Repeated measures ANOVA was utilized to compare changes of radiological parameters and SRS-22 outcome scores from preoperative to early postoperative and 2 years postoperatively between groups (coronal balance vs. imbalance). Statistical analyses were performed using the Statistical Package for Social Sciences Software (version 19.0; SPSS, Chicago, IL, USA), with p<0.05 considered statistically significant.

## 3. Results

### 3.1. Demographic Data

This study included 17 male and 119 female patients with a mean age of 15.3±3.4 years. The mean follow-up period was 28.5±12.3 months. There were 96 and 40 patients with Lenke 1 and 2 type curves, respectively. Of the patients, 46, 50, and 40 showed lumbar curve modifier A, B, and C, respectively. All of the patients showed a right-sided main thoracic curve. The number of levels fused was 9.8±4.6 (Table 1).

### 3.2. Radiological Assessment

**Figure 1:**
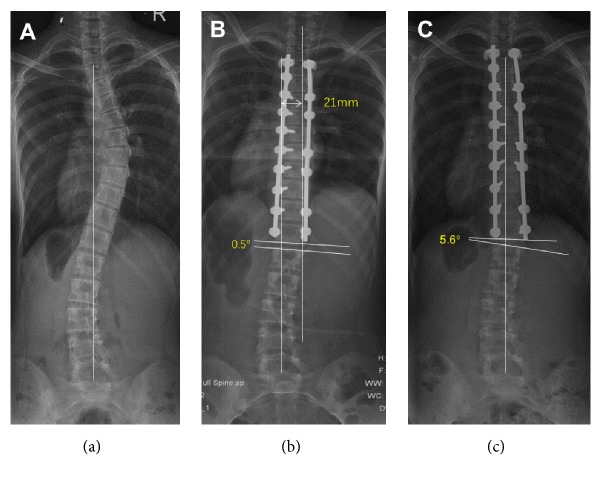
A 13-year-old female with Lenke 1AN adolescent idiopathic scoliosis underwent selective posterior thoracic fusion from T3 to T12. The preoperative coronal balance of -3mm (a) worsened to 21mm immediately after surgery (b), but recovered to 2mm at the final follow-up (c). LIV disc angle increased from 0.5° in the early postoperative period to 5.6° at the final follow-up.

Of the patients with an A lumbar curve modifier, coronal imbalance was noticed in 10 patients preoperatively, in 16 patients immediately postoperatively, and in no patient at final follow-up, suggesting that all the patients showed spontaneous correction during follow-up (Table 2). Comparisons of radiological parameters in the postoperative period according to early postoperative decompensation are described in Table 3. No differences except C7-CSVL were found in the early postoperative period. However, greater LIV-CSVL (-13.7mm vs. -5.3mm, p=0.023) and LIV disc angle (6.7° vs. 1.2°, p=0.032) were found in the imbalanced group at the final follow-up. LIV-CSVL (-3.4mm vs. -13.7mm, p=0.020) and LIV disc angle (0.9° vs. 6.7°, p=0.019) increased significantly in the imbalanced group at the final follow-up compared with those in the early postoperative period. A representative case is illustrated in Figure 1.

**Figure 2:**
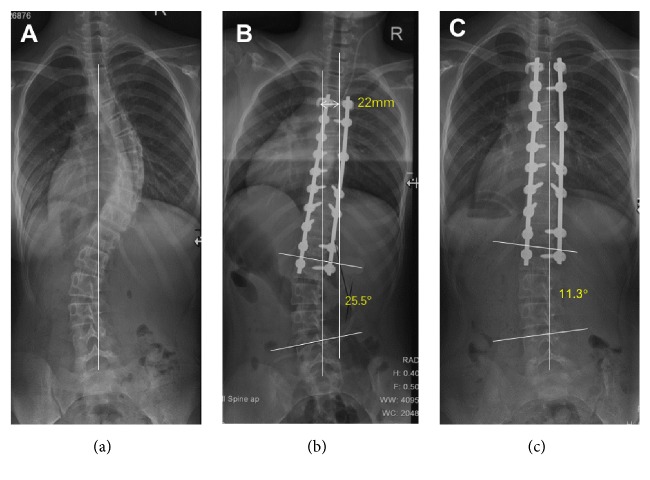
A 15-year-old female with Lenke 1BN adolescent idiopathic scoliosis underwent selective posterior thoracic fusion from T4 to L1. The preoperative coronal balance of -2mm (a) worsened to 22mm immediately after surgery (b), but recovered to 1mm at the final follow-up (c). The lumbar angle decreased from 25.5° in the early postoperative period to 11.3° at the final follow-up.

Of the patients with a B lumbar curve modifier, coronal imbalance was noticed in 10 patients preoperatively, in 15 patients immediately postoperatively, and in no patient at final follow-up (Table 2). A greater TL/L curve was found in the imbalanced group in the early postoperative period. However, no differences were found at the final follow-up (Table 4, Figure 2).

**Figure 3:**
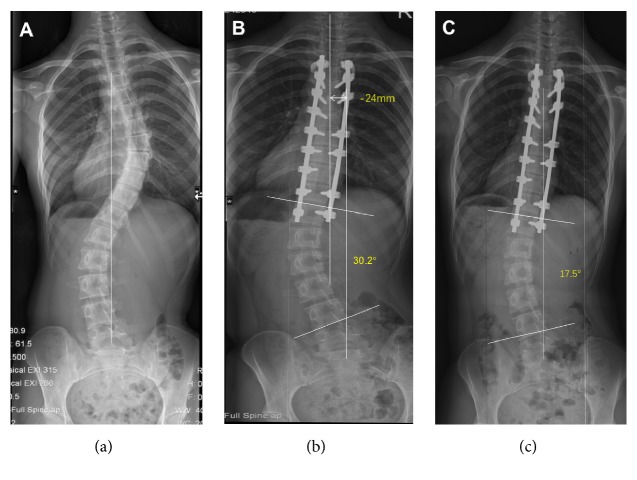
A 15-year-old female with Lenke 1CN adolescent idiopathic scoliosis underwent selective posterior thoracic fusion from T4 to T12. The preoperative coronal balance of 6mm (a) worsened to -24mm immediately after surgery (b), but recovered to -3mm at the final follow-up (c). The lumbar angle decreased from 30.2° in the early postoperative period to 17.5° at the final follow-up.

Of the patients with a C lumbar curve modifier, coronal imbalance was noticed in 13 patients preoperatively, in 17 patients immediately postoperatively, and in 2 patients at final follow-up (Table 2). Although the TL/L curve showed a statistical difference between the 2 groups in the early postoperative period (25.5° vs. 13.7°, p<0.01), this difference disappeared in the final follow-up with decrease of TL/L curve in the imbalanced group (Table 5, Figure 3).

### 3.3. Clinical Outcomes

SRS-22 scores showed no difference between the coronal balanced and imbalanced groups preoperatively and the final follow-up. The self-image domain score of the imbalanced group was a little lower than that of balanced group during the early postoperative period, although the difference was not statistically significant (3.74 vs. 4.12, p=0.052) (Table 6).

## 4. Discussion

The main goal of STF is maintenance of a balanced spine, spontaneous correction of compensatory thoracic curves, and saving more mobile lumbar segments [8]. The use of PS constructs allows perfect correction of the main thoracic curve. However, coronal decompensation is one of the significant problems that occur after STF in AIS [9, 10]. For Lenke type 1/2 curves, especially with a C lumbar modifier, there is increased risk of preoperative and postoperative coronal imbalance, while in curves with an A or B lumbar modifier, the distribution of preoperative and postoperative coronal decompensation is still lacking.

In addition, spontaneous improvement of coronal balance after STF has also been noted and investigated. It was proposed that patients with Lenke 1C tended to be decompensated to the left preoperatively (40%) [3]. In one recent study, postoperative coronal balance improvement after STF was frequent (8/10) for Lenke 1/2 C AIS [4]. These results were supported by our findings. In our study, 13 (29.5%) patients with Lenke 1/2 C curves were decompensated greater than 20 mm preoperatively, 17 (42.5%) patients immediately postoperatively, and 2 (5%) patients at the final follow-up. As for patients with Lenke 1/2 A or B curves, the incidence of coronal imbalance preoperatively is slightly lower (21.7% and 20. 0%, respectively). And it is also common for patients with Lenke 1/2 A or B curves experiencing favorable spontaneous correction of coronal balance on long term follow-up.

The causative factors of postoperatively detected coronal imbalance after STF have been a frequent focus of investigation. However, the factors associated with the spontaneous correction of postoperative coronal imbalance have still been poorly studied. The immediately postoperative coronal balance [4] and trunk shift [6] were reported to have significantly negative correlation to postoperative coronal balance remodeling for patients with Lenke 1/2 C type curves. It was reported that a fixation more distal to stable vertebrae would shift the coronal balance further to the left postoperatively [4].

As for patients with Lenke 1/2 A type curves, no parameters but C7-CSVL showed significant difference between the two groups in the early postoperative period. However, both LIV-CSVL and LIV disc angle increased during follow-up in the imbalanced group. This means that coronal balance in Lenke 1/2 A–AIS might be maintained by the development of “adding-on.” Adding-on is a common complication in AIS patients after AIS correction surgery, and patients with type A modifier may be more prone to postoperative distal adding-on than those with type B and C modifiers [11, 12]. In one study, adding-on developed less frequently in Lenke 2A-AIS patients with a larger clavicle angle at follow-up after posterior thoracic fusion surgery, implying that distal adding-on can help compensate for shoulder imbalance [13]. It was also reported that more decompensated coronal balance immediately after surgery was found in the adding-on group in patients with severe and rigid scoliosis who underwent posterior spinal fusion surgery [14]. Although the clinical significance of adding-on during a long follow-up period remains to be illustrated [15], our findings indicated that adding-on might be a compensatory mechanism for coronal balance in patients with Lenke 1/2 A type curves with type A modifier.

For patients with type B or C modifier, spontaneous correction of lumbar curve may attribute to the improvement of coronal imbalance. In our study, the thoracolumbar/lumbar curve angle showed a different changing pattern between the 2 groups of C type. Although it did not change in the balanced group, it decreased during follow-up in the imbalanced group. This means that coronal balance might be maintained by the spontaneous correction of thoracolumbar/lumbar curves. The influence of the lumbar curve on the postoperative behavior of coronal balance has been previously studied [16]. It was proposed that the overcorrection of the thoracic curve relative to the lumbar curve was a risk factor for postoperative decompensation in some patients after STF [17]. In one study, overcorrection was defined as the correction more than the major curve's flexibility and it would cause problems in the compensatory curves unless the compensatory is flexible and it corrects totally in bending radiographies, suggesting that lumbar curve magnitude and flexibility have statistically significant impact on postoperative coronal balance [18]. In another study, after overcorrection of Lenke type 1 curve, coronal balance did not show any significant difference between early postoperative period and last follow-up, and it implied that selective fusion with overcorrection in Lenke 1A could be applied when the lumbar curve could be corrected at the preoperative bending radiograph with no preoperative coronal decompensation [19].

In our study, only two patients with C modifier showed persistent decompensated coronal balance during the final follow-up. Similar SRS-22 outcome scores were found between the coronal imbalanced and balanced groups during the preoperative, early postoperative, and the last follow-up period. Only slight higher self-image scores were shown in the balanced group in the early postoperative period. In one study, patients presented with widely deviated compensatory lumbar curves underwent STF and the patients who developed postoperative coronal imbalance had slightly inferior SRS-24 results at latest follow-up (2-16 years) [20]. In another study, AIS patients with preoperative coronal decompensation after STF showed no significant differences in SRS-22 outcome scores between groups that were postoperatively balanced or persistently decompensated [21]. We assumed that the decompensation occurred early postoperatively may have little influence on patients' clinical outcome, but the clinical outcome of the persistently decompensated patients in our study needs to be further studied with a larger cohort and a longer follow-up.

### 4.1. Limitations

First, this study was a retrospective study with a relative small number of subjects with coronal imbalance. Second, the follow-up period was relatively short and we could not evaluate the clinical and radiographic outcomes of the coronal imbalance and adding-on developed at the final follow-up.

## 5. Conclusions

In conclusion, coronal imbalance was frequently detected immediately after STF in Lenke 1 or 2 AIS patients, with type C modifier slightly higher than A or B, but it was mostly corrected spontaneously. Distal adding-on may help compensate for coronal imbalance in patients with type A modifier, while spontaneous correction of lumbar curve attributes to the improvement of coronal imbalance in patients with type B or C modifier.

## Figures and Tables

**Table 1:** General data of patients.

Characteristics	N or mean (SD/range)
Age (years old)	15.3±3.4
Gender (F/M)	119/17
Follow-up (months)	28.5±12.3
Fused segments	9.8±4.6
Lenke classification	
1	96
2	40
Lumbar curve modifier	
A	46
B	50
C	40

**Table 2:** Incidence of preoperative, early postoperative, and final follow-up coronal imbalance.

	preoperative	early postoperative	final follow-up
A (N=46)	10 (21.7%)	16 (34.8%)	0 (0.0%)
B (N=50)	10 (20.0%)	15 (30.0%)	0 (0.0%)
C (N=40)	13 (29.5%)	17 (42.5%)	2 (5.0%)

**Table 3:** Comparisons of postoperative radiological parameters by early postoperative decompensation in lumbar curve modifier A.

	Early postoperative period			Final follow-up		
	Coronal Imbalance	Coronal balance	P value	Coronal Imbalance	Coronal balance	P value
	(N=16)	(N=30)		(N=16)	(N=30)	
PT curve (°)	9.7±7.5	10.8±6.8	0.604	10.2±9.8	10.5±7.9	0.908
MT curve (°)	15.4±8.3	13.7±9.6	0.536	16.2±7.3	13.9±8.5	0.345
TL/L curve (°)	17.3±8.7	15.9±6.6	0.531	17.5±9.0	17.0±10.2	0.865
AVT-MT (mm)	3.7±6.8	3.5±8.4	0.932	4.5±7.8	3.8±9.4	0.792
AVT-TL/L (mm)^*∗*^	4.5±7.6	6.5±9.5	0.452	6.7±9.3	7.4 ±9.5	0.804
C7-CSVL (mm) ^*∗*^	22.4±15.5	8.6±12.3	0.001	11.6±7.5^#^	8.1±5.9	0.079
UIV tilt (°)	3.8±6.8	4.3±5.9	0.790	3.7±7.3	4.6±5.0	0.615
LIV tilt (°)	6.5±11.2	5.4±8.7	0.705	6.9±10.8	6.0±9.3	0.761
LIV-CSVL (mm) ^*∗*^	3.4±8.9	2.5±7.6	0.711	13.7±15.5^#^	5.3±9.3	0.023
LIV disc angle (°)	0.9±1.5	0.7±3.4	0.815	6.7±9.2^#^	1.2±7.8	0.032

Data represents mean and standard deviation.

*∗*Negative means the measurement is located left to the reference line (CSVL).

#p<0.05, when the parameters at the final follow-up are compared with those in early postoperative period.

PT: proximal thoracic; MT: main thoracic; TL/L: thoracolumbar/lumbar; AVT: apical vertebral translation; CSVL: central sacral vertical line; UIV: upper instrumented vertebra; LIV: lower instrumented vertebra.

**Table 4:** Comparisons of postoperative radiological parameters by early postoperative decompensation in lumbar curve modifier B.

	Early postoperative period			Final follow-up		
	Coronal Imbalance	Coronal balance	P value	Coronal Imbalance	Coronal balance	P value
	(N=15)	(N=35)		(N=15)	(N=35)	
PT curve (°)	13.8±6.9	10.6±9.4	0.206	14.6±7.3	11.3±11.0	0.256
MT curve (°)	15.3±10.5	10.9±9.8	0.135	16.3±12.4	13.2±8.8	0.293
TL/L curve (°)	19.5±9.8	12.6±6.9	0.004	13.5±7.8^#^	11.4±5.0	0.235
AVT-MT (mm)	3.5±7.1	2.7±6.5	0.681	4.3±8.2	3.6±9.3	0.788
AVT-TL/L (mm)^*∗*^	7.6±8.6	6.7±5.3	0.637	8.3±9.3	7.3 ±6.5	0.647
C7-CSVL (mm) ^*∗*^	22.9±7.9	9.0±6.5	0.001	12.6±9.0^#^	8.8±7.6	0.110
UIV tilt (°)	5.4±6.7	4.9±4.5	0.746	6.0±7.7	5.3±6.5	0.727
LIV tilt (°)	7.6±9.8	5.7±6.5	0.399	8.3±7.6	6.1±8.0	0.337
LIV-CSVL (mm) ^*∗*^	3.8±9.1	3.1±6.7	0.750	2.7±4.9	2.4±5.0	0.835
LIV disc angle (°)	0.3±1.5	0.1±2.1	0.720	0.5±3.4	0.2±4.4	0.801

Data represents mean and standard deviation.

*∗*Negative means the measurement is located left to the reference line (CSVL).

#p<0.05, when the parameters at the final follow-up are compared with those in early postoperative period.

PT: proximal thoracic; MT: main thoracic; TL/L: thoracolumbar/lumbar; AVT: apical vertebral translation; CSVL: central sacral vertical line; UIV: upper instrumented vertebra; LIV: lower instrumented vertebra.

**Table 5:** Comparisons of postoperative radiological parameters by early postoperative decompensation in lumbar curve modifier C.

	Early postoperative period			Final follow-up		
	Coronal Imbalance	Coronal balance	P value	Coronal Imbalance	Coronal balance	P value
	(N=17)	(N=23)		(N=17)	(N=23)	
PT curve (°)	15.4±8.7	16.5±7.6	0.662	16.8±11.3	16.3±9.6	0.876
MT curve (°)	17.9±7.9	18.3±8.4	0.875	19.3±5.8	20.3±6.3	0.599
TL/L curve (°)	25.5±12.3	13.7±7.2	0.001	15.7±7.8^#^	14.3±6.5	0.525
AVT-MT (mm)	4.5±9.4	3.7±10.9	0.803	8.3±10.2	7.6±12.3	0.845
AVT-TL/L (mm)^*∗*^	-15.3±12.3	-13.5±14.2	0.667	-12.7±10.9	-10.0 ±11.5	0.442
C7-CSVL(mm) ^*∗*^	-23.5±9.7	-10.9±5.6	0.001	-13.2±6.8^#^	-11.5±6.7	0.419
UIV tilt (°)	6.7±7.7	5.8±9.4	0.741	7.8±9.3	7.0±11.3	0.807
LIV tilt (°)	8.0±9.2	9.3±10.7	0.679	12.4±11.4	13.5±13.2	0.777
LIV-CSVL (mm) ^*∗*^	-5.4±7.6	-3.8±4.5	0.392	-4.5±5.0	-3.9±4.6	0.686
LIV disc angle (°)	1.1±7.3	0.8±6.5	0.888	0.9±7.8	0.7±9.8	0.943

Data represents mean and standard deviation.

*∗*Negative means the measurement is located left to the reference line (CSVL).

#p<0.05, when the parameters at the final follow-up are compared with those in early postoperative period.

PT: proximal thoracic; MT: main thoracic; TL/L: thoracolumbar/lumbar; AVT: apical vertebral translation; CSVL: central sacral vertical line; UIV: upper instrumented vertebra; LIV: lower instrumented vertebra.

**Table 6:** SRS-22 scores of patients in the preoperative and early postoperative period.

Domain	Preoperative			Early postoperative			Final follow-up		
	Coronal	Coronal	*P* value	Coronal	Coronal	*P*	Coronal	Coronal	*P* value
	imbalance	balance		imbalance	balance	value	imbalance	balance	
Function/activity	3.78±1.21	3.86±0.84	0.672	3.93±0.93	4.02±1.12	0.636	4.01±1.02	4.12±0.86	0.506
Pain	3.92±1.10	3.73±0.95	0.338	4.02±0.86	3.84±0.76	0.210	3.94±0.93	3.81±0.72	0.367
Self-image	3.82±0.96	4.04±1.22	0.346	3.74±0.74	4.12±1.03	0.052	4.02±0.91	4.13±0.97	0.520
Mental health	4.23±1.37	4.39±0.93	0.448	4.30±0.86	4.41±0.96	0.509	4.42±0.88	4.53±0.76	0.447
Satisfaction	3.56±1.20	3.76±0.94	0.323	3.66±1.09	3.84±1.22	0.395	3.98±1.23	4.05±0.96	0.714
Total	3.73±1.13	3.90±0.75	0.322	3.92±1.12	3.98±0.98	0.746	4.05±0.96	4.09±0.79	0.794

Data represents mean and standard deviation.

## Data Availability

The data used to support the findings of this study are available from the corresponding author upon request.

## Abbreviations

AIS: Adolescent idiopathic scoliosis
STF: Selective thoracic fusion
LIV: Lowermost instrumented vertebra
CSVL: C7 plumb line and central sacral vertical line
SV: Stable vertebra
UIV: Uppermost instrumented vertebra
PT: Proximal thoracic
MT: Main thoracic
TL: Thoracolumbar/lumbar
AVT: Apical vertebral translation.
